# Selective cell death of latently HIV-infected CD4^+^ T cells mediated by autosis inducing nanopeptides

**DOI:** 10.1038/s41419-019-1661-7

**Published:** 2019-05-29

**Authors:** Gang Zhang, Brian T. Luk, Xiaoli Wei, Grant R. Campbell, Ronnie H. Fang, Liangfang Zhang, Stephen A. Spector

**Affiliations:** 1 Division of Infectious Diseases, Department of Pediatrics, University of California San Diego, La Jolla, CA USA; 2 Department of NanoEngineering and Moores Cancer Center, University of California San Diego, La Jolla, CA USA; 3 Rady Children’s Hospital, San Diego, CA USA

**Keywords:** Macroautophagy, HIV infections

## Abstract

Despite significant advances in the treatment of human immunodeficiency virus type-1 (HIV) infection, antiretroviral therapy only suppresses viral replication but is unable to eliminate infection. Thus, discontinuation of antiretrovirals results in viral reactivation and disease progression. A major reservoir of HIV latent infection resides in resting central memory CD4^+^ T cells (T_CM_) that escape clearance by current therapeutic regimens and will require novel strategies for elimination. Here, we evaluated the therapeutic potential of autophagy-inducing peptides, Tat-Beclin 1 and Tat-vFLIP-α2, which can induce a novel Na^+^/K^+^-ATPase dependent form of cell death (autosis), to kill latently HIV-infected T_CM_ while preventing virologic rebound. In this study, we encapsulated autophagy inducing peptides into biodegradable lipid-coated hybrid PLGA (poly lactic-co-glycolic acid) nanoparticles for controlled intracellular delivery. A single dose of nanopeptides was found to eliminate latent HIV infection in an in vitro primary model of HIV latency and ex vivo using resting CD4^+^ T cells obtained from peripheral blood mononuclear cells of HIV-infected patients on antiretroviral with fully suppressed virus for greater than 12 months. Notably, increased LC3B lipidation, SQSTM1/p62 degradation and Na^+^/K^+^-ATPase activity characteristic of autosis, were detected in nanopeptide treated latently HIV-infected cells compared to untreated uninfected or infected cells. Nanopeptide-induced cell death could be reversed by knockdown of autophagy proteins, ATG5 and ATG7, and inhibition or knockdown of Na^+^/K^+^-ATPase. Importantly, viral rebound was not detected following the induction of the Na^+^/K^+^-ATPase dependent form of cell death induced by the Tat-Beclin 1 and Tat-vFLIP-α2 nanopeptides. These findings provide a novel strategy to eradicate HIV latently infected resting memory CD4^+^ T cells, the major reservoir of HIV latency, through the induction of Na^+^/K^+^-ATPase dependent autophagy, while preventing reactivation of virus and new infection of uninfected bystander cells.

## Introduction

At present, an estimated 37 million people live with human immunodeficiency virus type-1 (HIV) infection worldwide^1^. Despite the tremendous success of antiretroviral therapy (ART) in suppressing the virus and changing HIV from an invariably fatal disease to a chronic illness, current treatment strategies have failed to eradicate the virus and achieve a virologic cure. Additionally, despite prolonged virologic suppression, the discontinuation of ART in most cases leads to the rapid return of viremia^2–4^. The difficulty in eradicating HIV from latent reservoirs has changed the focus of much research to be directed towards achieving a “functional cure”. As part of a functional cure strategy, numerous investigators are attempting strategies to reactivate HIV from latent reservoirs (shock) followed by killing the virus. However, the “shock and kill” approach has, to date, been unsuccessful because methods attempted have failed to reactivate latent virus^5^.

The primary reservoirs of HIV latently infected cells are thought to be long-lived, resting central memory CD4^+^ T cells (T_CM_), which are established early in infection, harbor integrated proviral DNA, and fail to produce replication-competent virus^6–8^. These latently infected cells are not targeted by the immune system and ART is ineffective in eradicating the virus. Although the precise mechanisms that promote the long-term survival of HIV latently infected T_CM_ are largely unknown, it is likely that cell death pathways are critical to cell survival, and that anti-apoptotic proteins and modulation of autophagy play an important role in prolonged cell survival.

Macroautophagy (referred to here as autophagy) is a critical cyto-adaptive response to environmental stresses including starvation, ischemia, cancer and infection^9,10^. The hallmark of autophagy is a double-membraned autophagosome that engulfs bulk cytoplasm and cytoplasmic organelles, such as mitochondria and endoplasmic reticulum^11^. The accumulation of autophagosomes and autophagic proteins within a cell represents the failure of autophagy to rescue the cell from toxic stress^12^. Although the major role of autophagy is cell survival and maintenance of cellular homeostasis, the over induction of autophagy can lead to autophagic cell death^13,14^. During initial HIV infection of host cells, there is an induction of autophagy and HIV uses autophagy-related proteins (ATG) to promote its own replication^15^. During permissive infection, HIV down-regulates autophagy to prolong cell survival. In the case of CD4^+^ T cells, autophagy is dysfunctional and most infected cells die^16^. However, infection of macrophages and microglial cells leads to a balance between the levels of autophagy required for cell survival and low-level viral replication without killing the cell.

In addition to extending cell survival, autophagy plays a central role in the degradation and elimination of intracellular pathogens^10,17–19^. Our laboratory has had a particular interest in examining the role of autophagy in HIV pathogenesis^20–23^, and identified that the induction of autophagy inhibits HIV replication and promotes the degradation of viral proteins^23–26^. Once HIV establishes a productive infection, HIV Nef binds Beclin 1 resulting in mTOR activation, TFEB phosphorylation and cytosolic sequestration, and the inhibition of autophagy^23^. These findings help to explain how HIV modulates autophagy to promote cell survival and viral persistence, and further establishes Beclin 1 as an important target to eliminate latent reservoirs of HIV. The region of Beclin 1 binding to Nef has been mapped to an 18 aa conserved region^27^. In conjunction with the laboratory of Dr. Beth Levine, we showed that a Tat-Beclin 1 fusion peptide consisting of the Tat transduction domain and the identified region of Beclin 1 that binds Nef is a potent inducer of autophagy^27^. Additionally, we showed that pre-treatment of macrophages with this Tat-Beclin 1 peptide inhibits HIV infection. Similar proteins that inhibit autophagy have been found in other viruses. One of these, viral FLIP (vFLIP) present in Kaposi’s sarcoma-associated herpesvirus, herpesvirus saimiri and molluscum contagiosum virus, was found to inhibit autophagy and cell death by preventing Atg3 from binding and processing LC3, a critical protein in autophagosome biogenesis^28^.

Liu et al. have described a novel form of autophagic cell death that they have termed “autosis” which has unique morphologic features, depends on cellular Na^+^/K^+^-ATPase, and occurs during treatment with autophagy inducing peptides, starvation and hypoxia^29,30^. In a previous study, we found that autophagy inducing peptides, Tat-Beclin 1 and Tat-vFLIP-α2, exhibit robust anti-HIV activity through induction of autophagy^31^. Both autophagy-inducing peptides induce cell death through autosis that is dependent on alteration of Na^+^/K^+^-ATPase. Of interest, we found that intracellular delivery of Tat-Beclin 1 and Tat-vFLIP-α2 peptides by lipid-coated hybrid PLGA (poly lactic-co-glycolic acid) nanoparticles can preferentially induce autosis and selectively kill chronically infected macrophages. In this study, we further evaluated the potential of these nanoformulated autophagy-inducing peptides (nanopeptides) to kill latently HIV-infected central memory CD4^+^ T cells (HIV-T_CM_). Our findings demonstrate that nanopeptides can induce a unique Na^+^/K^+^-ATPase dependent form of cell death that has the potential to eliminate latently HIV-infected T_CM_ without reactivation of HIV replication, and protect bystander cells.

## Results

### Nanopeptides preferentially kill CD4^+^ T memory cells with latent infection

We have previously shown the ability of lipid-coated hybrid PLGA nanoparticles loaded with Tat-vFLIP-α2 peptides to kill selectively HIV-infected macrophages^31^. However, the most common reservoir of HIV is thought to reside in resting central memory CD4^+^ T cells (T_CM_)^32,33^. Thus, our initial experiments were designed to demonstrate the potential of these nanopeptides to preferentially kill HIV-T_CM_. Because the frequency of latently HIV-infected CD4^+^ T cells in infected persons on fully suppressive ART is extremely low, our laboratory has adapted a primary in vitro latency model of HIV-infected resting CD4^+^ central memory T cells for screening novel anti-HIV latency altering agents^34^. In this model, HIV-T_CM_ lack both cell surface activation and cell cycle markers, do not synthesize DNA, do not proliferate, and contain an average of 1 copy per cell of integrated HIV DNA. Importantly, HIV can be reactivated from these cells using αCD3/αCD28-conjugated beads, phytohemagglutinin, M form (PHA-M) or interleukin 7. In the studies presented here, we further optimized this latency model through co-incubation with the HIV protease inhibitor, atazanavir, and the nucleoside reverse transcriptase inhibitor, tenofovir (Supplementary Fig. 1).

We formulated and characterized nanopeptides following our previous established protocol^31^. Using single step nanoprecipitation, the formulated PLGA nanoparticles loaded Tat-Beclin 1 (NP-Beclin 1) and Tat-vFLIP-α2 (NP-vFLIP-α2) had an average 127 nm and 147 nm in diameter, respectively (Supplementary Fig. 2). All of the formulated nanopeptides had more than 15% (wt:wt) loading yield, were stable in both PBS and water over the 96 h evaluation period, and did not exhibit any burst release of Tat-Beclin 1 or Tat-vFLIP-α2 in peptide release kinetics studies (Supplementary Fig. 2).

**Fig. 1:**
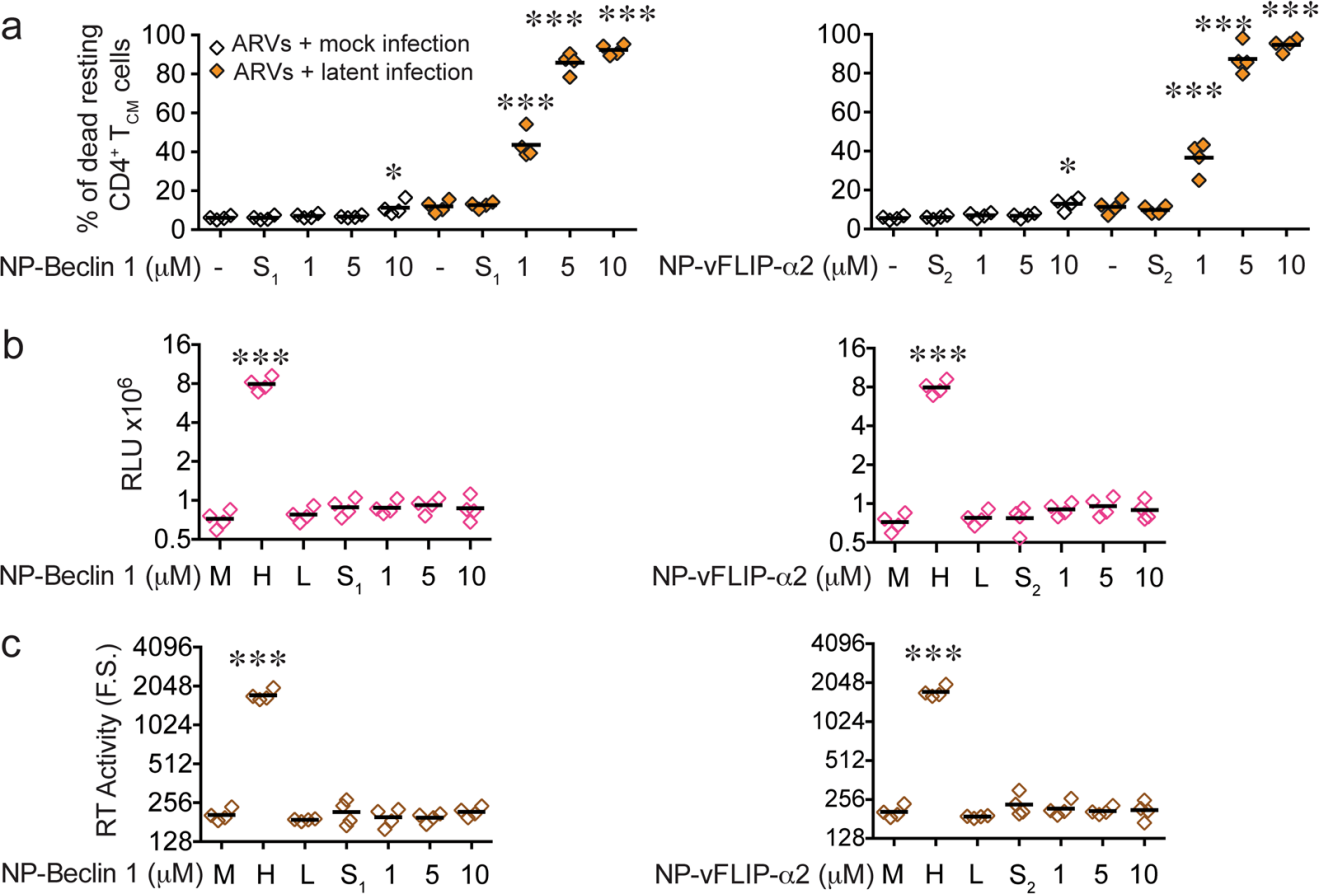
Nanopeptides preferentially kill latent primary HIV-T_CM_ cells. **a** At day 32 post infection (p.i.), latent HIV-T_CM_ cells were treated with increasing doses of NP-Beclin 1 or NP-vFLIP-α2 for 24 h. The cytotoxicity of NP-Beclin 1 and NP-vFLIP-α2 were measured by trypan blue staining. **b** The collected cell culture supernatants were incubated with TZM-bl cells. After 48 h, TZM-bl cells were measured for ß-galactosidase activity. **c** The collected cell culture supernatants were also tested for RT activity. Data are plotted from four different donors with means. M = Mock infection, H = 200 TCID_50_ HIV_NL-43_ virus, L = latent HIV-T_CM_ cells, NP-S_1_ = 10 μM nanoformulated Tat-Beclin-1 scrambled peptides, NP-S_2_ = 10 μM nanoformulated Tat-vFLIP-α2 scrambled peptides. RLU = relative luminescence units. F.S. = fluorescent signaling. *P < 0.05, ***P < 0.001

To assess the ability of our nanopeptides to induce preferential killing, NP-Beclin 1 and NP-vFLIP-α2 at increasing concentrations were incubated with HIV-T_CM_ and uninfected cells. After 24 h, both nanopeptides demonstrated a dose dependent killing of HIV-T_CM_. NP-Beclin 1 treatment resulted in 43.6, 85.7, and 92.3% cell death of HIV-T_CM_ at 1, 5, and 10 µM, respectively. Similarly, following NP-vFLIP-α2 treatment at the same concentrations, we observed 36.6, 87.3, and 94.6% killing of HIV-T_CM_ (Fig. 1a). In contrast, uninfected T_CM_ when treated under the same conditions at the highest concentration of 10 µM of nanopeptide resulted in 11.2 and 12.9% cell killing for NP-Beclin 1 or NP-vFLIP-α2, respectively.

### Nanopeptides induced selective killing does not reactivate HIV replication

We next evaluated whether nanopeptides would reactivate infectious virus following treatment of HIV-T_CM_. HIV infectious virus was assessed using the TZM-bl assay system^35^. After co-culture with TZM-bl for 48 h, we did not detect the presence of any infectious virus activated from the HIV-T_CM_ (Fig. 1b). These findings were further confirmed by quantification of viral reverse transcriptase (RT) activity in the collected supernatants from nanopeptide-treated HIV-T_CM_. No increase in RT activity was observed following administration of the nanopeptides (Fig. 1c), further confirming that our nanopeptides kill latently infected T_CM_ without reactivating HIV replication.

### Killing of latently infected CD4^+^ T cells is autophagy dependent

**Fig. 2:**
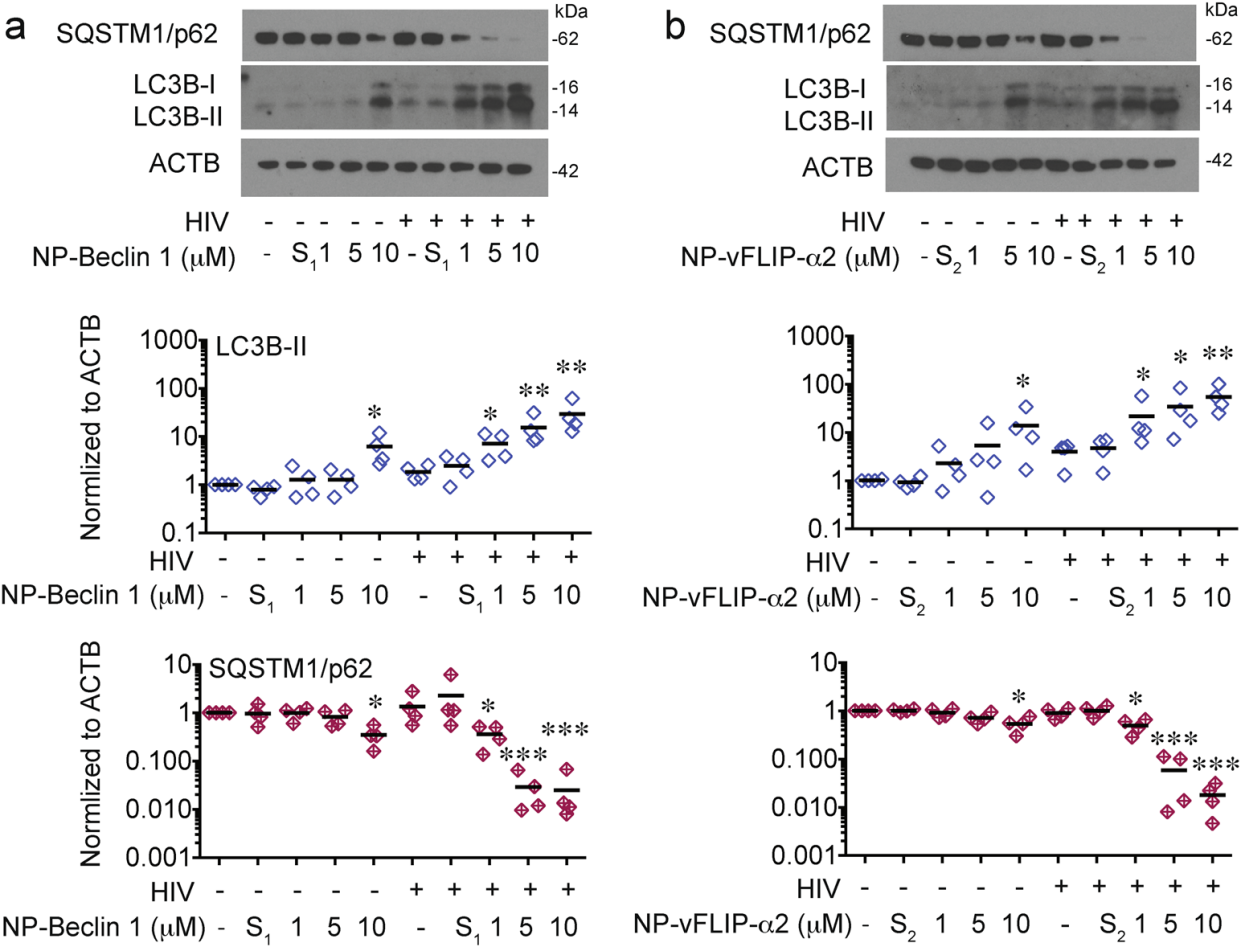
Nanopeptides induce enhanced autophagy in latent HIV-T_CM_ cells. **a**, **b** At day 32 p.i., HIV-T_CM_ cells were treated with NP-Beclin 1 and NP-vFLIP-α2, respectively for 24 h. Cell lysates were harvested for analysis of LC3B-II lipidation and SQSTM1/p62 degradation. Representative western blots are shown. Densitometric analysis are summarized from 4 different donors and normalized to loading control ACTB. NP-S_1_ = 10 μM nanoformulated Tat-Beclin-1 scrambled peptides, NP-S_2_ = 10 μM nanoformulated Tat-vFLIP-α2 scrambled peptides. *P < 0.05, **P < 0.01, ***P < 0.001

Based on our previous research, we suspected that the mechanism for the selective killing observed of HIV-T_CM_ would be dependent on an autophagy dependent form of cell death^31^. In these experiments, HIV-T_CM_ and uninfected T_CM_ were treated with increasing concentrations of nanopeptides. In HIV-T_CM_ for both NP-Beclin 1 and NP-vFLIP-α2, we observed a significant dose response increase in LC3B-II while sequestosome 1 (SQSTM1/p62) was significantly decreased indicating that autophagy was being induced and going to completion (*P* < 0.001; Fig. 2). In contrast for the HIV-uninfected T_CM_, except at the highest concentration of the nanopeptides (10 μM), there was little change in LC3B-II or SQSTM1/p62 (Fig. 2). However, even at the highest concentration, the decrease in SQSTM1/p62 in uninfected T_CM_ was significantly less than that observed in HIV-T_CM_ (NP-Beclin 1, *P* < 0.01; NP-vFLIP-α2, *P* < 0.01).

**Fig. 3:**
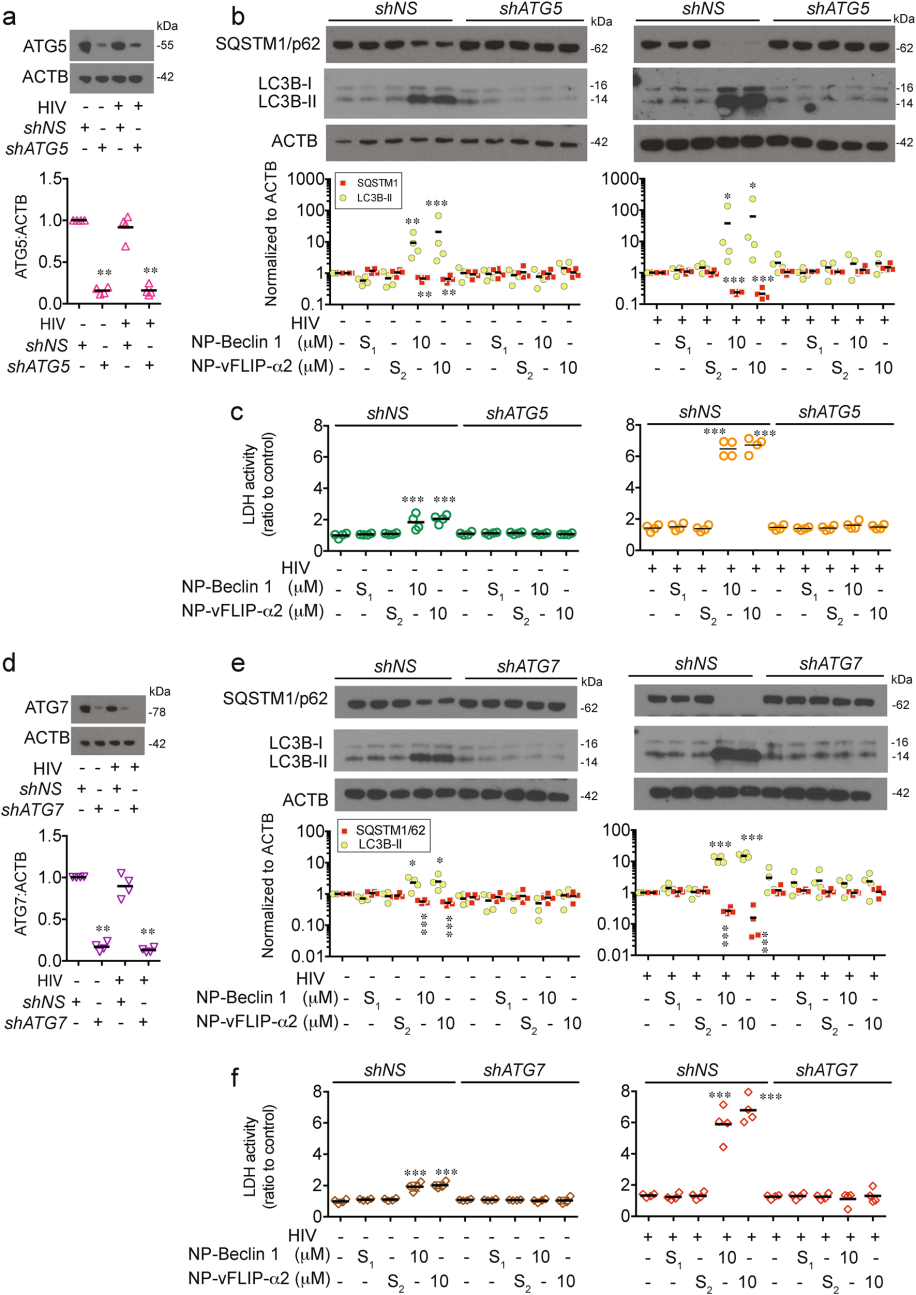
RNA interference of ATG5 and ATG7 inhibits nanopeptide-induced autophagy dependent cell death in latent HIV-T_CM_ cells. **a**, **d** Lentiviral sh*ATG5* and sh*ATG7* transduced latently infected resting CD4^+^ T cells were tested for knockdown efficiency by western blot. **b**, **e** sh*ATG5* and sh*ATG7* transduced latent CD4^+^ T_CM_ cells were challenged with 10 μM NP-Beclin 1 or 10 μM NP-vFLIP-α2 for 24 h. Autophagy was evaluated in cell lysates by western blot. **c**, **f** Cytotoxicity of NP-Beclin 1 and NP-vFLIP-α2 was measured in cell culture supernatants. Densitometric analyses are summarized from four different donors and normalized to loading control ACTB with means. NP-S_1_ = 10 μM nanoformulated Tat-Beclin-1 scrambled peptides, NP-S_2_ = 10 μM nanoformulated Tat-vFLIP-α2 scrambled peptides. *P < 0.05, **P < 0.01, ***P < 0.001

To further confirm the importance of autophagy in NP-Beclin 1 and NP-vFLIP-α2 mediated cell death, we assessed the effect of *ATG5* and *ATG7* silencing. Knockdown of *ATG5* and *ATG7* reversed nanopeptide-induced cell death (Fig. 3), and inhibited LC3B-II lipidation and SQSTM1/p62 degradation further confirming that NP-Beclin 1 and NP-vFLIP-α2 induced preferential cell death is through an autophagy dependent mechanism.

**Fig. 4:**
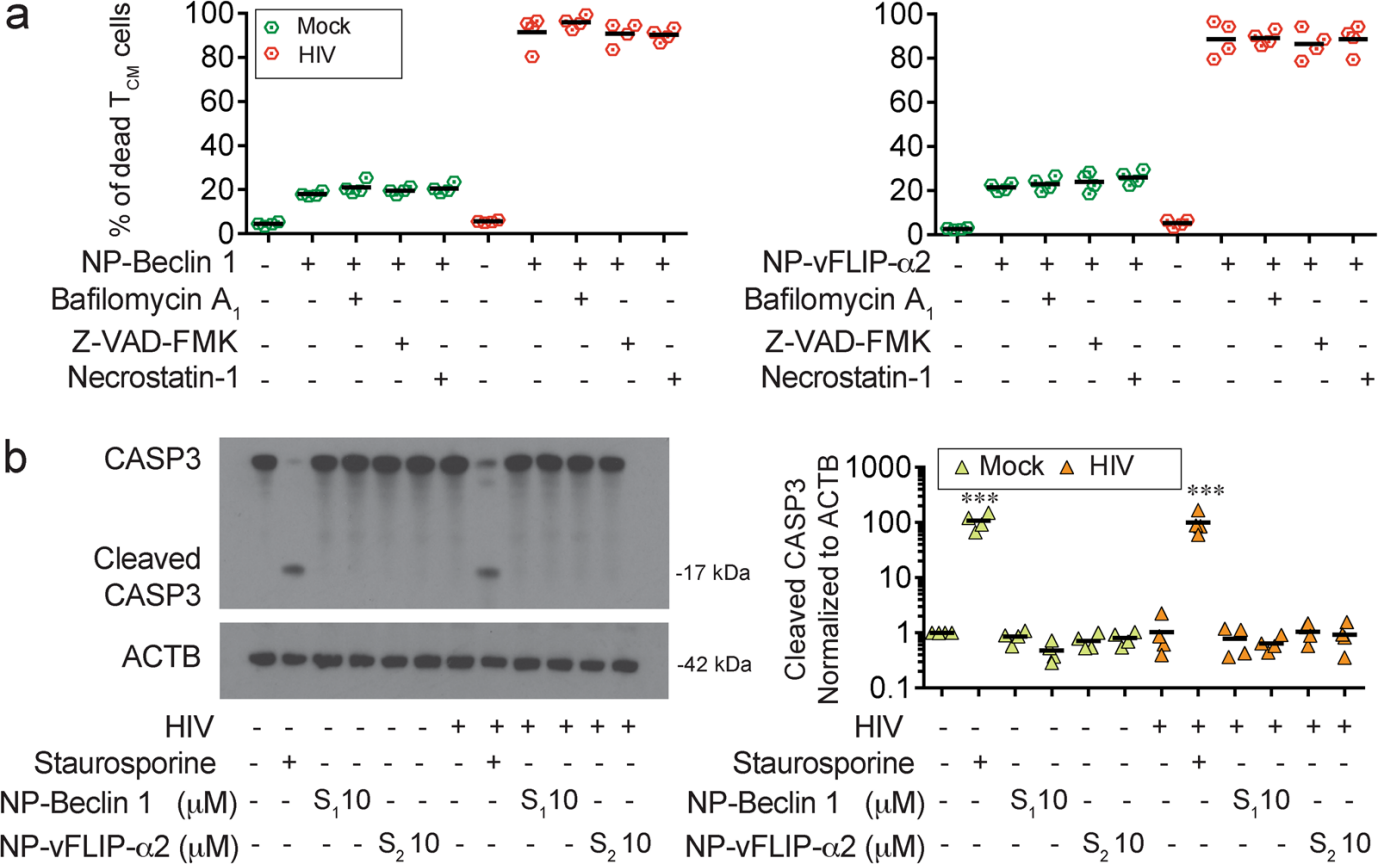
Nanopeptides induce caspase-independent cell death. **a** HIV-T_CM_ cells were pretreated with 20 μM Z-VAD-FMK, 50 μM necrostatin-1, and 200 nM bafilomycin A_1_ for 2 h, and further challenged with 10 μM NP-Beclin 1 or 10 μM NP-vFLIP-α2 for an additional 24 h. The cell culture supernatants were collected for LDH cytotoxicity assay. **b** Cleaved CASP3 was analyzed in the harvested cell lysates by western blot. All densitometric analyses are summarized from four different donors and normalized to loading control ACTB with means. NP-S1 = 10 μM nanoformulated Tat-Beclin 1 scrambled peptides, NP-S2 = 10 μM nanoformulated Tat-vFLIP-α2 scrambled peptides. *P < 0.05, **P < 0.01, ***P < 0.001

Having demonstrated an important role of autophagy in our nanopeptides induced killing of HIV-T_CM_, it was important to exclude other potential causes of cell death including apoptosis and necroptosis. In these experiments, HIV-T_CM_ were treated with the specific pharmacologic inhibitors of interest followed by exposure to either NP-Beclin 1 or NP-vFLIP-α2 nanopeptides. We used the pan-caspase inhibitor z-VAD-FMK for assessing apoptosis and the RIPK1 inhibitor necrostatin-1 for testing necroptosis. Treatment with either inhibitor, however, had no effect on nanopeptide induced cell death (Fig. 4). To further verify that the observed cell death is not due to apoptosis or necroptosis, we assessed HIV-T_CM_ and T_CM_ cultures for the presence of cleaved caspase (CASP)3. Cleaved CASP3, the active form of CASP3^36,37^, is induced by CASP8 and CASP9, and is considered a key initiator of apoptosis, pyroptosis and necroptosis. Consistent with our previous findings, the expression of cleaved CASP3 in HIV-T_CM_ or uninfected T_CM_ was unchanged following treatment with NP-Beclin 1 and NP-vFLIP-α2, further supporting that the observed cell death was not related to apoptosis and necroptosis.

### Tat-Beclin 1 and Tat-vFLIP-α2 induce a Na^+^/K^+^-ATPase dependent form of cell death, autosis

**Fig. 5:**
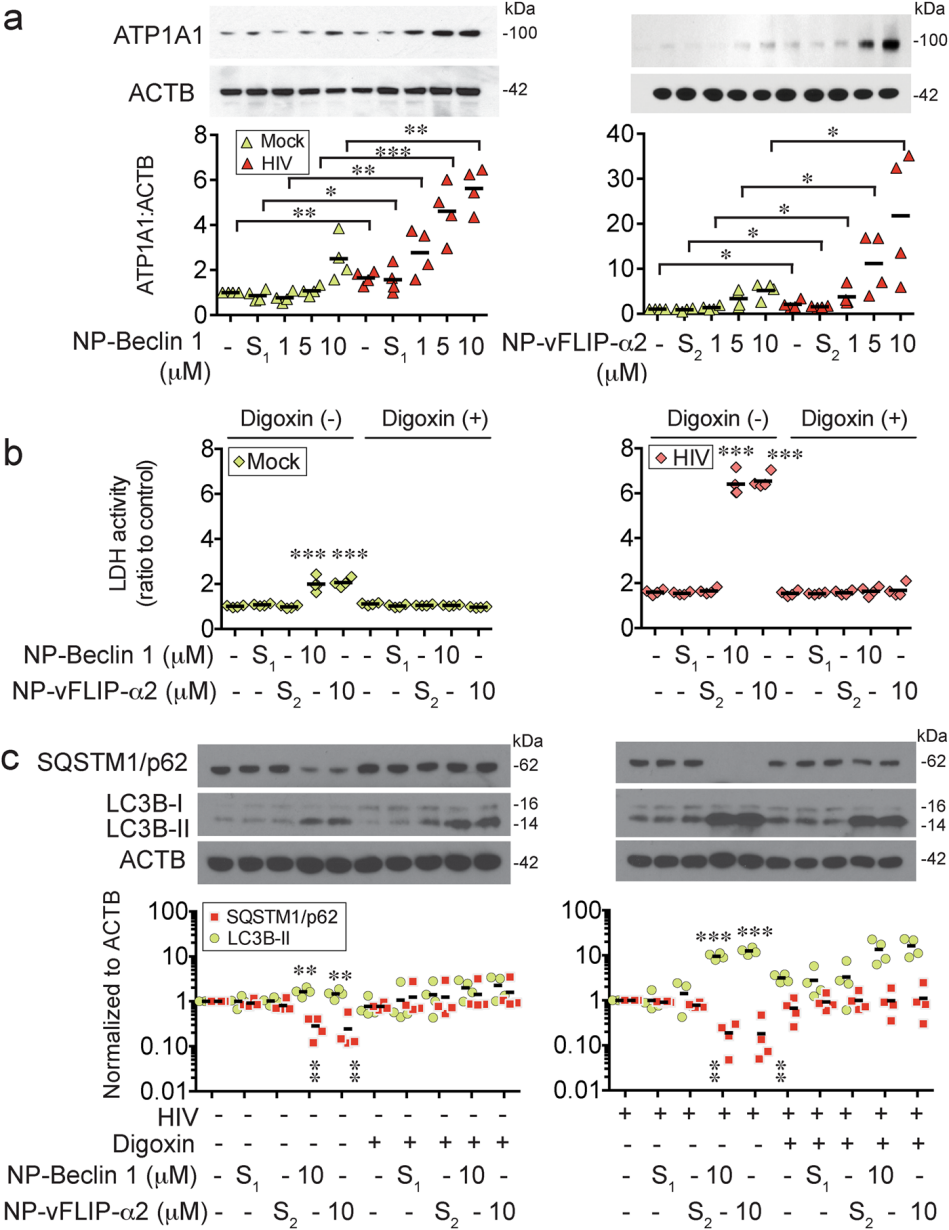
NP-Beclin 1 and NP-vFLIP-α2 kills latent HIV-T_CM_ cells through a Na^+^/K^+^-ATPase dependent mechanism. **a** HIV-T_CM_ were incubated with increasing concentrations of NP-Beclin 1 or NP-vFLIP-α2 for 24 h. The collected cell lysates were tested for Na^+^/K^+^-ATPase-α1 subunit (ATP1A1) expression by western blot. **b** After pretreating with 50 nM digoxin for 2 h, latent HIV-T_CM_ were incubated with NP-Beclin 1 or NP-vFLIP-α2 for 24 h. The cytotoxicity was monitored by LDH assay. **c** Autophagy activity was analyzed in collected cell lysates by western blot. All densitometric analyses are summarized from four different donors and normalized to loading control ACTB with means. NP-S_1_ = 10 μM nanoformulated Tat-Beclin-1 scrambled peptides, NP-S_2_ = 10 μM nanoformulated Tat-vFLIP-α2 scrambled peptides. *P < 0.05, **P < 0.01, ***P < 0.001

In previous studies performed by our lab and others, Tat-Beclin 1 and Tat-vFLIP-α2 have been shown to induce a Na^+^/K^+^-ATPase dependent form of cell death that has been designated as autosis^29–31,38^. To determine if autosis is responsible for the selective killing of HIV-T_CM_, we evaluated the expression of Na^+^/K^+^-ATPase in nanopeptide-treated HIV-T_CM_ (Fig. 5a). At increasing concentrations of both nanopeptides, we observed an increasing expression of the alpha 1 subunit of Na^+^/K^+^-ATPase (ATP1A1), which is the critical catalytic component for activating Na^+^/K^+^-ATPase. We also found that both nanopeptides induced a dose dependent increase of ATP1A1 in the mock-infected T_CM_. However, comparing with the same dose of nanopeptide induced ATP1A1 in the HIV-T_CM_ cells, NP-Beclin 1 induced a mean of 72.1, 76.7, and 55.4% less expression of ATP1A1 in mock-infected cells; similarly, NP-vFLIP-α2 induced 64.6, 69.1, and 75% less expression of ATP1A1 in the mock infected cells.

**Fig. 6:**
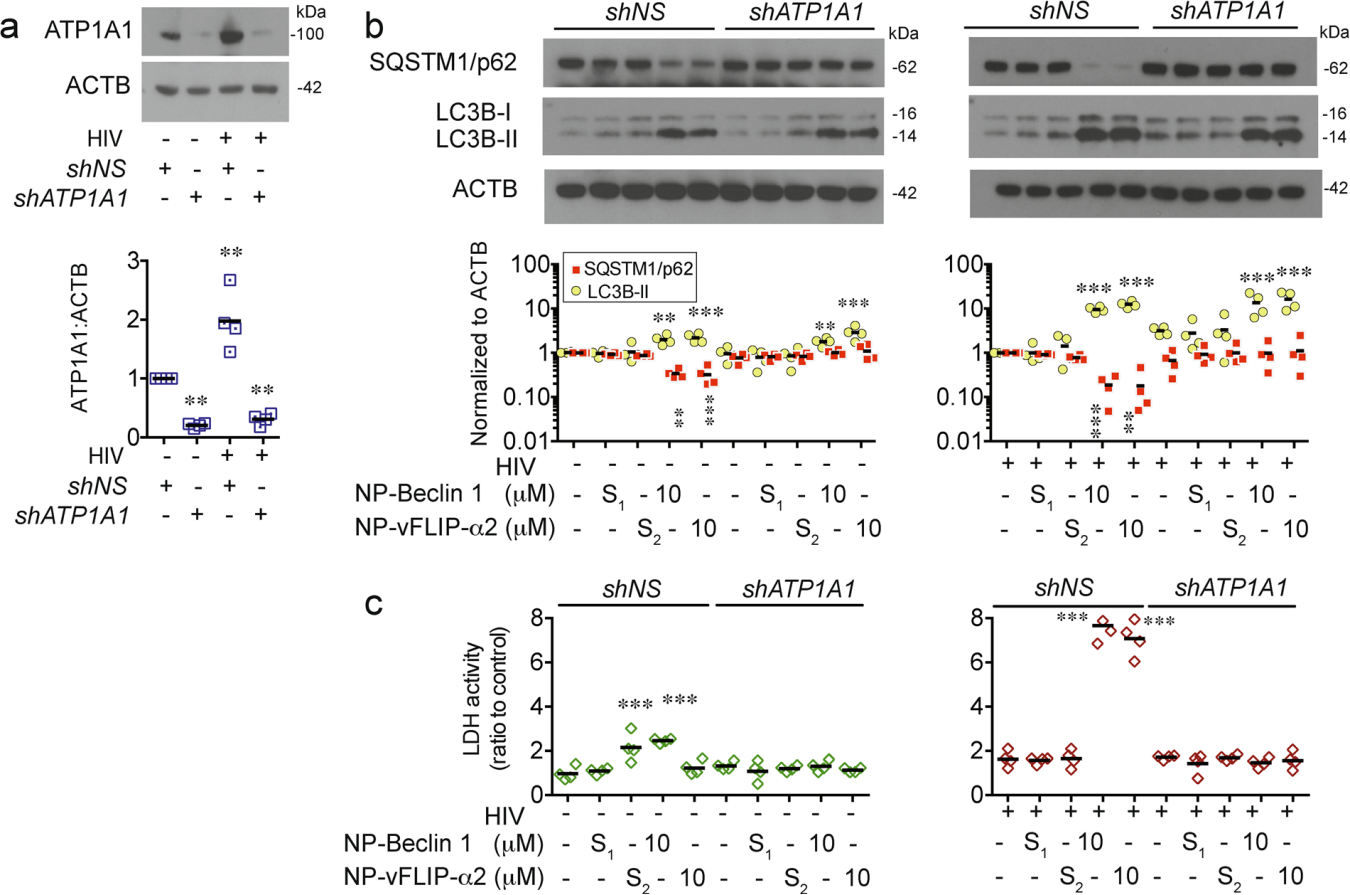
Knockdown of Na^+^/K^+^-ATPase inhibits NP-Beclin 1 and NP-vFLIP-α2 induced killing of latent HIV-T_CM_ cells. **a** HIV HIV-T_CM_ cells were transduced with sh*ATP1A1* for knockdown of Na^+^/K^+^-ATPase. The knockdown efficiency was evaluated by western blot in cell lysates. **b** sh*ATP1A1* transduced latent HIV-T_CM_ cells were treated with 10 μM NP-Beclin 1 or 10 μM NP-vFLIP-α2 for an additional 24 h. The effect of *shATP1A1* transduction was tested by western blot in cell lysates. **c** Cytotoxicity was measured by LDH assay. All densitometric analyses are summarized from four different donors and normalized to loading control ACTB with means. NP-S_1_ = 10 μM nanoformulated Tat-Beclin-1 scrambled peptides, NP-S_2_ = 10 μM nanoformulated Tat-vFLIP-α2 scrambled peptides. **P < 0.01, ***P < 0.001

To further establish that induction of the Na^+^/K^+^-ATPase is the mechanism of cell death, we pre-treated T_CM_ cultures with digoxin, a known inhibitor of Na^+^/K^+^-ATPase, 2 h prior to exposure to either NP-Beclin 1 or NP-vFLIP-α2. After 24 h, digoxin treated cultures demonstrated a marked reduction in cell death (Fig. 5b, c). Digoxin treated cultures also showed a marked reduction in autophagic flux as demonstrated by the absence of SQSTM1/p62 degradation. A similar effect was observed in experiments where ATP1A1 was knocked down using shRNA (Fig. 6). In total, these findings confirm that Na^+^/K^+^-ATPase is essential to Tat-Beclin 1 and Tat-vFLIP-α2 induced T_CM_ cell death.

### HIV infection increases Na^+^/K^+^-ATPase in CD4^+^ T memory cells with acute and latent infection

**Fig. 7:**
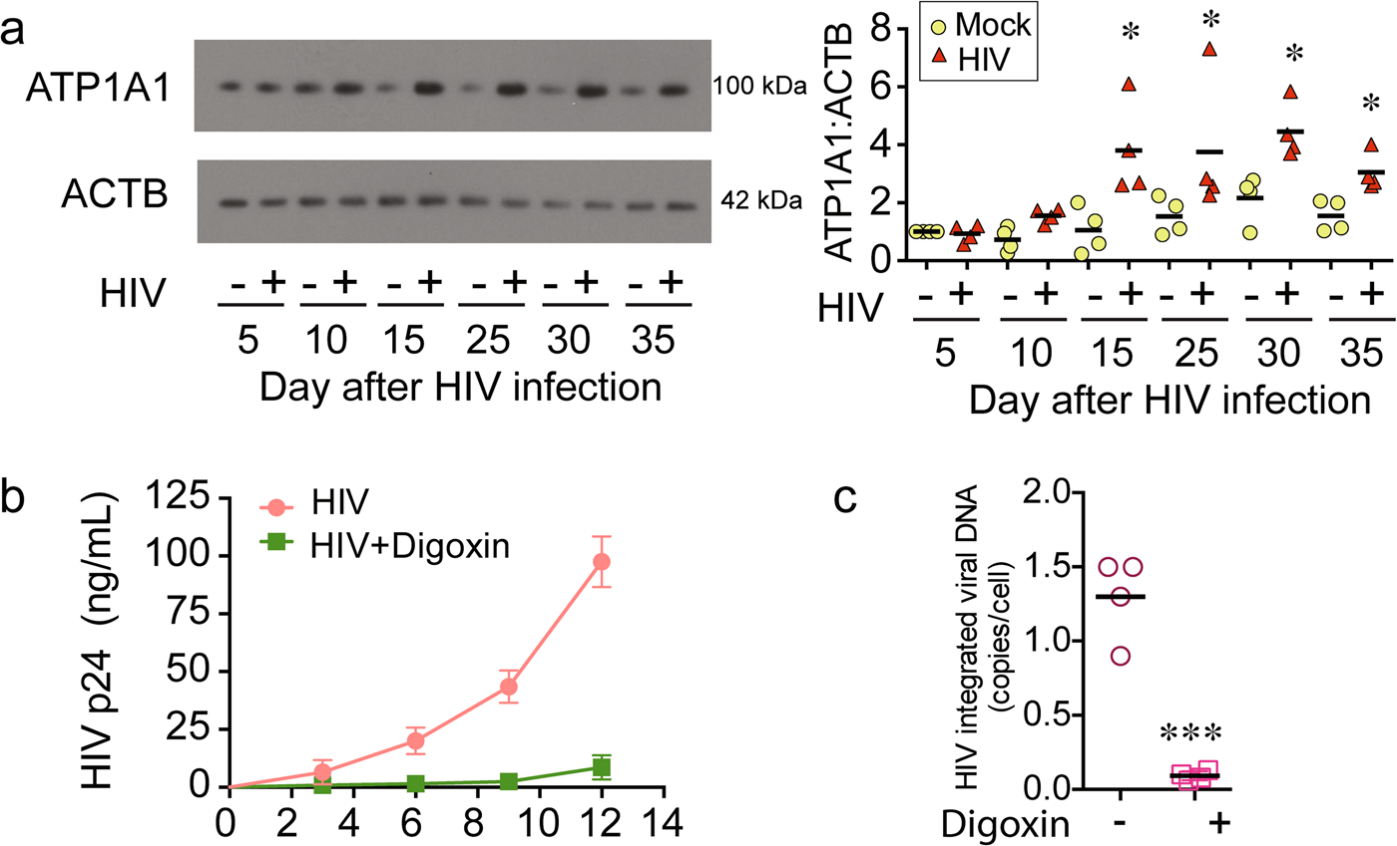
HIV infection increases Na^+^/K^+^-ATPase in CD4^+^ resting memory T cells. **a** ATP1A1 expression level was measured by western blot of cell lysates of CD4^+^ T memory cells. **b** CD4^+^ T cells were pretreated with 50 nM digoxin for 2 h followed by infection with HIV_NL-43_ (M.O.I = 0.1). The infected CD4^+^ T cells were incubated with 50 nM digoxin for 12 days. The cell culture supernatants were tested for HIVp24 by ELISA. **c** At day 12 p.i, digoxin treated CD4^+^ T cells under went magnetic negative selection to enrich for central memory CD4^+^ T cells. The purified CD4^+^ T memory cells were treated with 50 nM digoxin, 100 nM atazanavir, and 200 nM tenofovir for another 20 days. The harvested cells were measured for integrated HIV DNA using Alu-gag QPCR. All analyses are summarized from four different donors and normalized to loading control ACTB with mean. *P < 0.05, ***P < 0.001

Recently, cardiac glycosides/aglycones which inhibit Na^+^/K^+^-ATPase were identified to inhibit HIV replication^39–41^. Combined with our findings presented here, we hypothesized that cells latently infected with HIV might have increased Na^+^/K^+^-ATPase activity that contributes to the generation of latency. For these experiments, HIV-T_CM_ were established and evaluated for the presence of ATP1A1. Of interest, as the replication of virus in T_CM_ progressed to latency, the amount of ATP1A1 detected steadily increased over 30 days (Fig. 7a). To explore the potential function of Na^+^/K^+^-ATPase in generating HIV-T_CM_, we incubated Na^+^/K^+^-ATPase inhibitor digoxin with HIV-infected CD4^+^ T cells, and maintained digoxin treatment throughout the 30 days as the infection progressed to latency. As the infection progressed, the production of HIV p24 was significantly lower in the digoxin treated cells (Fig. 7b). When treated and untreated cultures reached latency, those cells treated with digoxin had a mean of 0.2 copies of proviral DNA per cells compared to 1.3 of HIV-T_CM_ controls (Fig. 7c).

### Nanopeptides preferentially kill ex vivo latent CD4^+^ T cells in patient blood

**Fig. 8:**
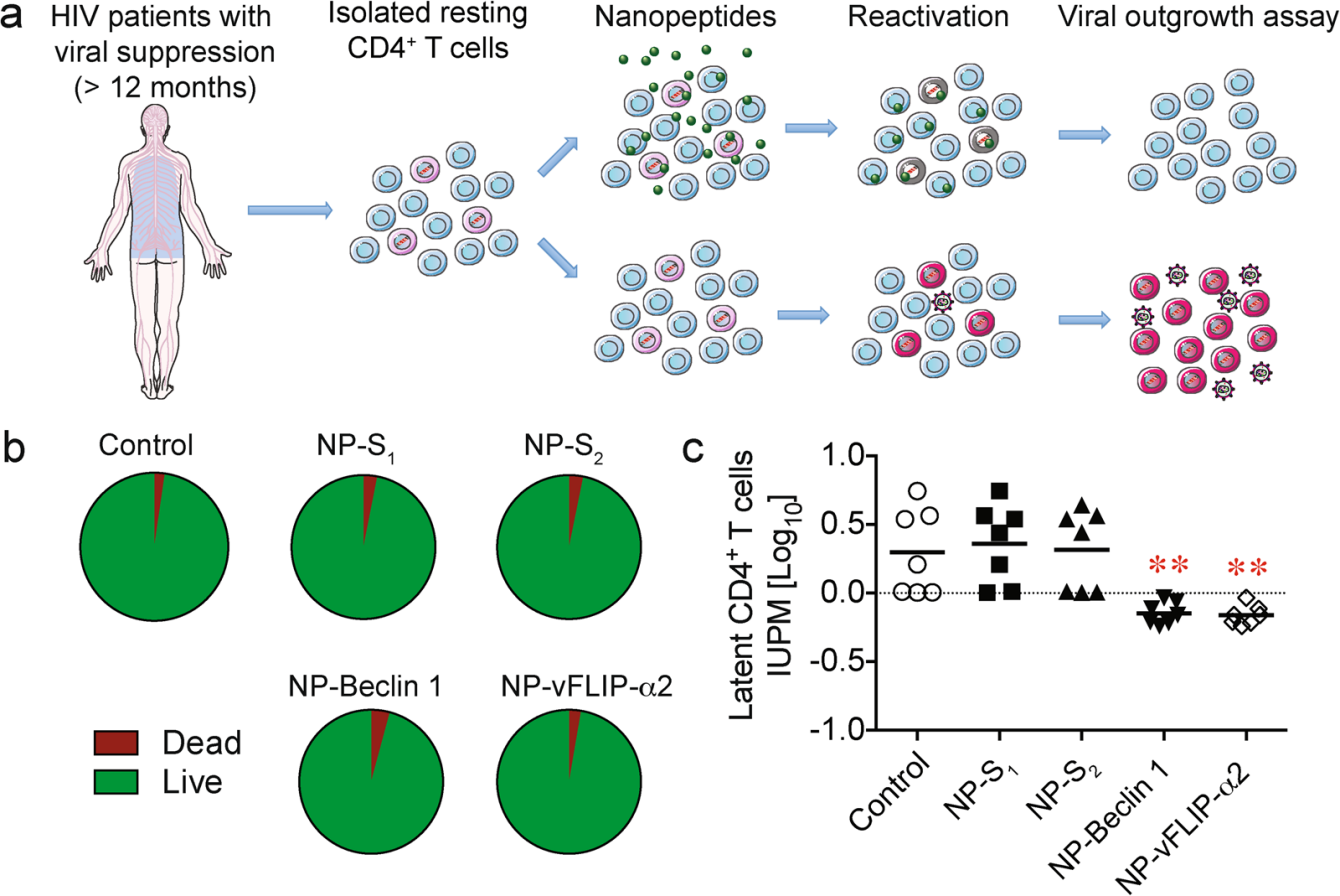
Nanopeptides reduce HIV latent infection in resting CD4^+^ T cells from HIV patients ex vivo. **a** HIV-infected patients who were receiving suppressive antiretroviral treatment, were virologically suppressed for >12 months (<20 copies HIV RNA/μL) and had >400 CD4^+^ cell/mL were recruited for blood donation. The purified resting CD4^+^ T cells were treated with 10 μM NP-Beclin 1 or 10 μM NP-vFLIP-α2 for 24 h, and then activated by PHA and γ-irradiation. Replication competent virus reactivated from CD4^+^ T cells was measured using a quantitative viral outgrowth assay (QVOA). **b** The cytotoxicity of NP-Beclin 1 and NP-vFLIP-α2 were tested with trypan blue staining in the purified patient resting CD4^+^ T cells after 24 h treatment. **c** The QVOA results were determined by HIVp24 ELISA and analyzed by maximum likelihood statistics. Data are summarized from seven different donors and plotted with means. IUPM = infectious units per million resting CD4^+^ T cells, NP-S_1_ = 10 μM nanoformulated Tat-Beclin-1 scrambled peptides, NP-S_2_ = 10 μM nanoformulated Tat-vFLIP-α2 scrambled peptides. **P < 0.01

To confirm that nanopeptides can preferentially kill HIV latently infected cells, we performed ex vivo studies on patient samples. PBMC were obtained from the blood of HIV-infected patients with viral suppression on ART, had undetectable viral loads defined as <20 copies HIV RNA/ml for at least 12 months and a CD4^+^ count of >400/mm^3^. Resting CD4^+^ T cells were isolated from PBMC and treated with nanopeptides for 24 h following the methods previously described by our laboratory^34^. Following treatment, there was no discernable increase in the number of dead cells in treated versus control cultures. This is not surprising given that latently infected cells comprise <10 per 10^6^ cells in vivo (Fig. 8a, b).

To identify the elimination of HIV latently infected cells that produce replication competent virus, the quantitative viral outgrowth assay (QVOA) was used^34^. Resting CD4^+^ T cells were isolated and treated with nanopeptides for 24 h, and CD4^+^ T cells were serially diluted and subjected to a limiting dilution quantitative outgrowth assay. Following a single treatment of NP-Beclin 1 and NP-vFLIP-α2, the amount of replication competent virus was reduced 70.8 and 71.8%, respectively (Fig. 8c), whereas the scrambled control nanopeptides had no effect. Thus, these findings provide further evidence that Tat-Beclin 1 and Tat-vFLIP-α2 have the potential to preferentially eliminate HIV latently infected cells with minimal cytotoxicity to uninfected cells in vivo.

### Inhibition of Na^+^/K^+^-ATPase prevents nanopeptide induced killing of ex vivo patient HIV latent CD4^+^ T cells

To determine the mechanism of nanopeptide mediated preferential killing of patient latently infected CD4^+^ T cells, we tested whether the decline in replication competent virus was dependent on the induction of Na^+^/K^+^-ATPase leading to autosis. For these studies, patient resting CD4^+^ T cells were incubated with digoxin for 2 h followed by treatment with nanopeptides for an additional 24 h at which time replication competent virus was assessed by QVOA. Following digoxin treatment, the anti-HIV and cell killing of latently infected cells of NP-Beclin 1 and NP-vFLIP-α2 was reversed and little difference was found in the quantity of infectious virus between the untreated and digoxin plus nanopeptide treated patient (Supplementary Fig. 3). Thus, these results confirm that our nanopeptides induce a Na^+^/K^+^-ATPase dependent cell death that can be reversed by inhibiting the Na^+^/K^+^-ATPase.

## Discussion

Considerable data support that HIV infection induces apoptosis in activated CD4^+^ T cells, impairing host immune function^42–46^. However, in some cells, HIV alters the transcriptional profile to promote the upregulation of anti-apoptotic proteins, leading to prolonged cell survival^47–51^. Additionally, during permissive infection, HIV Nef binds to Beclin 1, a key protein in autophagy, resulting in the down-regulation of autophagy that can lead to the prevention of autolysosomal degradation of the virus^23,52^. We have previously demonstrated that treatment of macrophages with Tat-Beclin 1 and Tat-vFLIP-α2 triggers the selective killing of HIV-infected macrophages while sparing uninfected macrophages^31^. However, a primary site of the HIV reservoir in persons on ART is believed to reside in long-lived, resting memory CD4^+^ T cells. Thus, in the current work, we have focused our research on this cell population. Our findings demonstrate both in an in vitro model and ex vivo studies that NP-Beclin 1 and NP-vFLIP-α2 can induce the selective killing of latent HIV-infected resting memory CD4^+^ T cells, while sparing uninfected cells and preventing new infection of bystander cells. Our research has further shown that the preferential killing of cells latently infected with HIV is due at least in part to the increased sensitivity of infected cells to alterations in the Na^+^/K^+^-ATPase, driving cells to a specific form of autophagy mediated cell death, autosis.

Numerous HIV cure strategies are currently under investigation. Although transplantation with CCR5Δ32 homozygous hematopoietic stem cells appears to have resulted in an HIV cure for one or two patients^53–55^, this strategy is impractical and has only been applied to patients with malignancies. Gene therapy approaches, on the other hand, are attempting to mimic this strategy using zinc finger nucleases to target CCR5 and more recently clustered regularly interspaced short palindromic repeats (CRISPR)/Cas-9^56-61^. Other gene editing approaches are attempting to use host restriction factors to engineer cells to resist HIV infection. Additional strategies aimed at reactivating HIV from latently infected cells using latency reversing agents in what has been termed the “shock and kill” approach whereby virus is reactivated and subsequently killed by antiretrovirals, and in some cases, combined with broadly neutralizing antibodies^5,62^. Numerous other approaches are attempting to modulate the immune response to HIV to control infection without the need for antiretroviral treatment in order to achieve what has been term a “functional cure”. Here, we have developed a novel approach that is capable of preferential killing HIV latently infected cells while sparing uninfected cells. This approach is based on several important principles: (1) Autophagy is essential for the maintenance of cellular homeostasis and persistence of HIV latent reservoirs; (2) Despite being an essential survival mechanism, excessive levels of autophagy are able to induce autophagy dependent cell death; (3) Autosis is an autophagy-dependent non-apoptotic form of cell death and can be triggered by autophagy-inducing peptides through the induction of Na^+^/K^+^-ATPase; and (4) The induction of autosis in addition to killing cells with replicating HIV, can kill HIV latently infected cells that are not undergoing viral replication without reactivation of virus.

Resting CD4^+^ T_CM_ cells, carrying replication-competent latent proviral DNA, are widely considered to remain at a quiescent status over years only to produce HIV upon activation. Although the role of autophagy in the establishment of HIV latent infection in resting CD4^+^ T_CM_ cells is unknown, many studies have shown that autophagy is essential for generating T memory cells and maintaining its immune function^63–65^. In memory CD8^+^ T cells, depletion of ATG5 and ATG7 directly induces cell death and impairs the memory phenotype^66^. Our previous studies have identified the antiviral effect of autophagy in controlling HIV infection in human primary macrophages and memory CD4^+^ T cells, through the formation of autophagosome and autolysosome mediated capture and degradation of viral proteins^24–26,31,34^. In this study, we further identify that autophagy-inducing peptides have the potential to eliminate HIV latent infection in T_CM_ cells using an autophagy-dependent mechanism.

To improve the translational potential of our approach, we have loaded the autophagy-inducing peptides into lipid-coated hybrid PLGA nanoparticles using biocompatible and biodegradable materials. Combining the unique features of liposomes and polymeric nanoparticles, our lipid-coated hybrid PLGA nanoparticles are capable of effective delivery of encapsulated cargos^67^. In this study, we successfully loaded Tat-Beclin 1 or Tat-vFLIP-α2 into the hybrid nanoparticles resulting in sustained release of autophagy-inducing peptides over 96 h. This lipid hybrid PLGA nanoformulation has been shown to extend the bioavailability of autophagy-inducing peptides in human macrophages for almost one week in our previous study^31^. Indeed, the hybrid nanoparticles have improved the intracellular bioavailability of our peptides to HIV-infected cells, and transformed the encapsulated peptide drug into biocompatible and translational drug candidate^68^. Recently, we developed a novel targeted version of the PLGA nanoparticles with surface coating of CD4^+^ T cell plasma membrane, which broadly recognizes HIV envelope protein, glycosylated protein 120 (gp120)^69^. This CD4^+^ T cell plasma membrane coated polymeric nanoparticle has high binding affinity to HIV gp120 leading to robust antiretroviral activity through neutralizing cell free HIV entering into CD4^+^ T cells and macrophages. This new formulation has the potential to improve the targeted killing of all HIVgp120 positive cells including chronically infected and reactivated latently infected cells.

In summary, we have identified that two nanopeptides, Tat-Beclin 1 and Tat-vFLIP-α2, targeting proteins critical to autophagy induce Na^+^/K^+^-ATPase and selectively kill HIV latently infected resting memory CD4^+^ T cells with little effect on uninfected cells. The preferential killing of latently infected cells is dependent on increased Na^+^/K^+^-ATPase as the induction of cell death can be reversed by digoxin, an inhibitor of Na^+^/K^+^-ATPase. These peptides when loaded into PLGA nanoparticles can be delivered and incorporated into HIV-infected cells, and are synthesized from FDA-approved material that will facilitate their ability to be used in humans. Thus, we believe that these peptides have great potential to be used as part of an overall strategy designed to eliminate HIV from infected persons.

## Materials and methods

### Ethics statement

Venous blood was obtained from HIV seronegative and HIV seropositive donors. The protocol was reviewed and approved by the Human Research Protections Program of the University of California, San Diego (Project 09-0660). Written informed consent was obtained from all blood donors.

### Preparation of NP-Beclin 1 and NP-vFLIP-α2

Autophagy-inducing peptides including Tat-Beclin 1 (RRRQRRKKRGY-GG-TGFEGDHWIEFTANFVNT), scrambled Tat-Beclin 1 (RRRQRRKKRGY-GG-WETAFGTTEHNIFFDNGV), Tat-vFLIP-α2 (RRRQRRKKRGY-GFVNLLFLVVE) and scrambled Tat-vFLIP-α2 (RRRQRRKKRGY-GFVNLAAAVVE), were synthesized in D-isomer sequence and obtained from New England Peptide, Inc. Autophagy-inducing peptides loaded lipid-coated PLGA nanoparticles were synthesized using single step nanoprecipitation. Poly (_D,L_-lactic-co-glycolic acid) PLGA (50:50, 0.67 dl/g, Pelham, AL) with ester-terminated and autophagy-inducing peptides were dissolved in organic solvent acetonitrile at 10 mg/ml. Soybean lecithin and DSPE-PEG_2000_-COOH (1,2-distearoyl-sn-glycero-3-phosphoethanolamine-N-carboxy (polyenylene glycol)2000) (Alabaster, AL) was mixed in chloroform at 20% of the PLGA polymer weight and air-dried as a lipid film. The dissolved PLGA/autophagy-inducing peptides organic solution was added into the lipid film under gentle stirring and further vortexed for 3 min. PLGA nanoparticles were then washed with 10 mM tris-HCl pH 8 buffer and loaded with autophagy-inducing peptides. The remaining acetonitrile and free polymers were removed by washing using an Amicon Ultra-4 centrifugal filter (Millipore, Billerica, MA) with a molecular weight cut-off of 10 kDa.

### Characterization of NP-Beclin 1 and NP-vFLIP-α2

The morphological characteristics of NP-Beclin 1 and NP-vFLIP-α2 were evaluated by JEOL Gatan transmission electron microscopy (TEM) using negative staining. The size, polydispersity index and surface zeta potential were measured by dynamic light scattering Malvern Zetasizer Nano ZS for each of nanoformulations. The peptide loading capacity was evaluated by Slide-A-Yzer MINI dialysis microtube with a molecular weight cutoff of 3.5 kDa (Thermofisher, Rockford, IL), and determined by the weight ratio of the peptide payload to the PLGA nanoparticles.

### Generation of HIV latently infected primary resting memory CD4^+^ T cells

HIV latently infected resting memory T cells were generated following our previously published protocol^34^. Briefly, resting memory CD4^+^ T cells from HIV-uninfected donors were suspended with RPMI 1640 medium with 10% fetal bovine serum supplemented with 29 nM CCL19 (PeproTech, Rocky Hill, NJ) for 48 h. Next, the CD4^+^ T cells were infected with HIV_NL4-3_ at a multiplicity of infection (M.O.I.) of 0.1, and incubated with 250 ng/mL staphylococcal enterotoxin B (Sigma, St.Louis, MO) and 25 U/mL IL-2 (Roche, Basel, Switzerland) for an additional 3 days. After removal of staphylococcal enterotoxin, cells were cultured with 25 U/mL IL-2 for another 9 days. Then, the central memory CD4^+^ T cells were purified from HIV-infected CD4^+^ T cells using negative magnetic isolation, and further cultured with 1 ng/mL IL-7 (PeproTech, Rocky Hill, NJ), 100 nM atazanavir and 200 nM tenofovir (Selleckchem, Houston, TX) for another 20 days.

### Quantitative viral out growth assay of HIV latent ex vivo CD4^+^ T in the isolated patient blood

Our laboratory has adapted the Quantitative viral out growth assay (QVOA) assay previously established by the Siliciano laboratory^34,70^. HIV-infected patients who were virologically suppressed on ART, had a viral load of <20 copies/mL for at least 12 months and had a CD4+ count of >400 cells/mm^3^ were recruited and obtained informed consent at University of California San Diego Mother-Child-Adolescent HIV Program clinic, and Peripheral blood mononuclear cells (PBMC) were isolated from collected venous blood using Ficoo-Paque^TM^ density centrifugation (GE Healthcare, Pittsburgh, PA). Resting CD4^+^ T cells were purified through negative magnetic cell isolation of human CD25, CD69 and anti-HLA-DR following the manufacturer’s protocols, and reactivated with gamma irradiation (5000R in Cs-source) and phytohemagglutinin (PHA-M, 1 μg/mL). The CD8^+^ T cell depleted PBMC were reactivated with PHA-M and used as feeder cells to co-culture with limiting dilution of resting CD4^+^ T cells for 3 weeks. The collected cell cultured supernatants were tested with HIV p24 antigen using by ELISA.

### Western blotting

The collected cell lysates were mixed with pierce lane marker reducing sample buffer (ThermoFisher Scientific, Waltham, MA) and boiled for 5 min to achieve protein denaturation. The protein samples were separated by ExpressPlus PAGE Gels and electrophoretic transferred to PVDF or nitrocellulose membrane. The targeted proteins were detected by primary anti-LC3B (Novus Biologicals, Littleton, CO), anti-ATP1A1, anti-ATG5, anti-ATG7 (Cell Signaling Technology, Danvers, MA) and anti-SQSTM1 antibodies (Abcam, Cambridge, MA). The horseradish peroxidase conjugated goat anti-mouse and anti-rabbit secondary antibodies were used to amplify the detected antigen.

### Cytotoxicity assay

The collected cell culture supernatants were co-incubated with LDH cytotoxicity assay reaction buffer following the manufacture’s protocol (Clontech, Mountain View, CA). The colorimetric results were quantified using a BioTek microplate reader at 490 nm wavelength. The dead cells were counterstained with 0.4% trypan blue solution and quantified by Nexcelom cell counter.

### TZM-bl HIV infectivity assay

TZM-bl HIV infectivity was tested following an established protocol^35^. Briefly, the collected cell culture supernatants were loaded into 96-well plates containing 50,000 TZM-bl cells per well in RPMI 1640 medium with 10% FBS, and incubated at 37 °C for 48 h. After washing, the incubated TZM-bl cells were measured by Beta-Glo assay kit (Promega, Madison, WI).

### Measurement of HIV infection

Genomic DNA from T_CM_ was isolated through the PureLink® Genomic DNA Kits (Invitrogen, Carlsbad, CA). Following the established protocol^71–73^, integrated viral DNA was measured with Alu-gag QPCR. The isolated genomic DNA from ACH-2 and 8E5 cell lines was used as HIV standards. The collected cell culture supernaturants were tested for HIVp24 ELISA (PerkinElmer, Waltham, MA) and RT activity (ThermoFisher, Carlsbad, CA) following the manufacture protocol.

### RNA interference

Short hairpin RNA lentiviral transduction particles kits were purchased from Sigma-Aldrich for silencing ATG5, ATG7 and ATP1A1 in primary central CD4+ T memory cells (ATG5-TRCN0000151963, ATG7-TRCN0000435480 and ATP1A1-TRCN0000424769). The lentiviral shRNA were transduced following the manufacture’s protocols. shRNA control vector was also obtained from Sigma-Aldrich used as non-specific targeting control (SHC002).

### Statistics

All results were assessed in GraphPad Prism 6.0 (GraphPad, La Jolla, CA). The normalized data including fold and ratio changes were transformed into log2 value to normalize the data. Two-tailed Student *t* test, ANOVA, Pearson correlation and Wilcoxon rank test were applied for statistical analysis. *P* values < 0.05 two-tailed were considered statistically significant.

## Supplementary information

Supplementary Fig 1, 2 and 3
