# Supplementary material for: Selective cell death of latently HIV-infected CD4+ T cells mediated by autosis inducing nanopeptides
Source: Cell Death Dis. 2019 May 29;10(6):419. doi: 10.1038/s41419-019-1661-7 (PMC6541658; doi:10.1038/s41419-019-1661-7)
Supplement: Supplementary file 1 — Supplementary Fig 1, 2 and 3 [file 41419_2019_1661_MOESM1_ESM.docx]

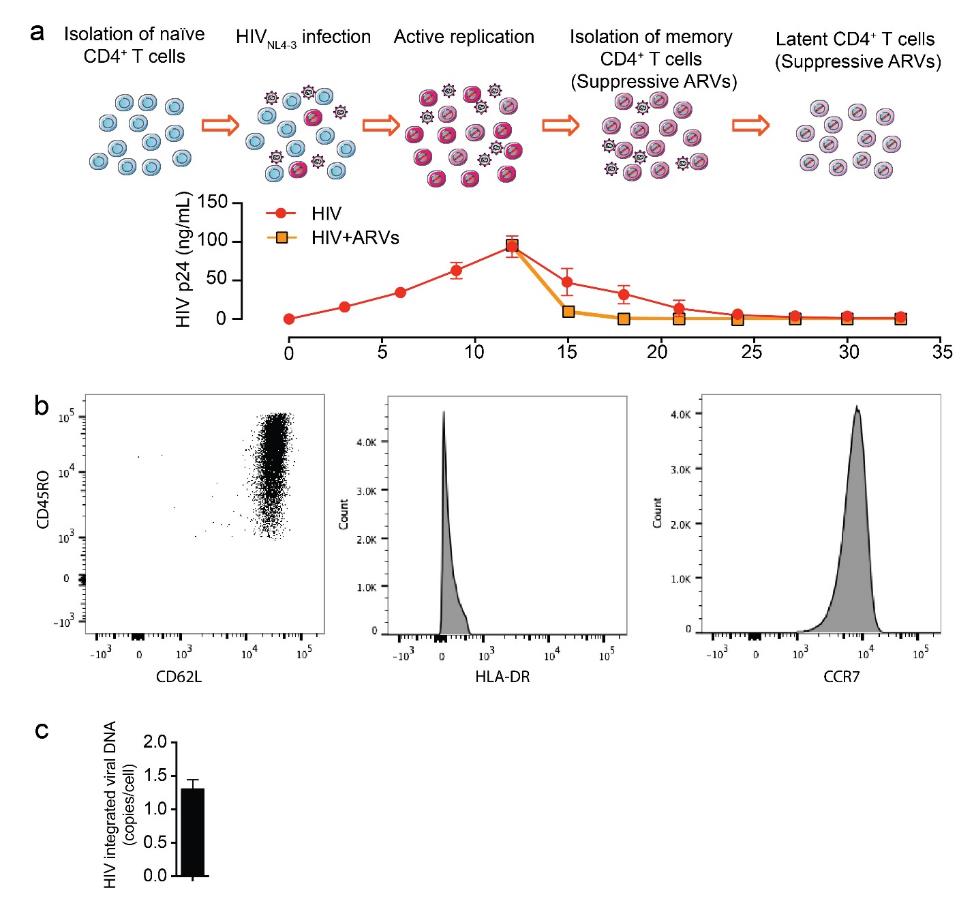


**Supplementary Fig. 1** Generation of central memory CD4^+^ T cells with latent HIV infection. **a** During generation of HIV latent infection, HIV replication was monitored using HIVp24 ELISA assay. **b & c** At day 32 p.i., latent HIV-T_CM_ were characterized by flow cytometry, and analyzed for integrated HIV DNA by QPCR.

**
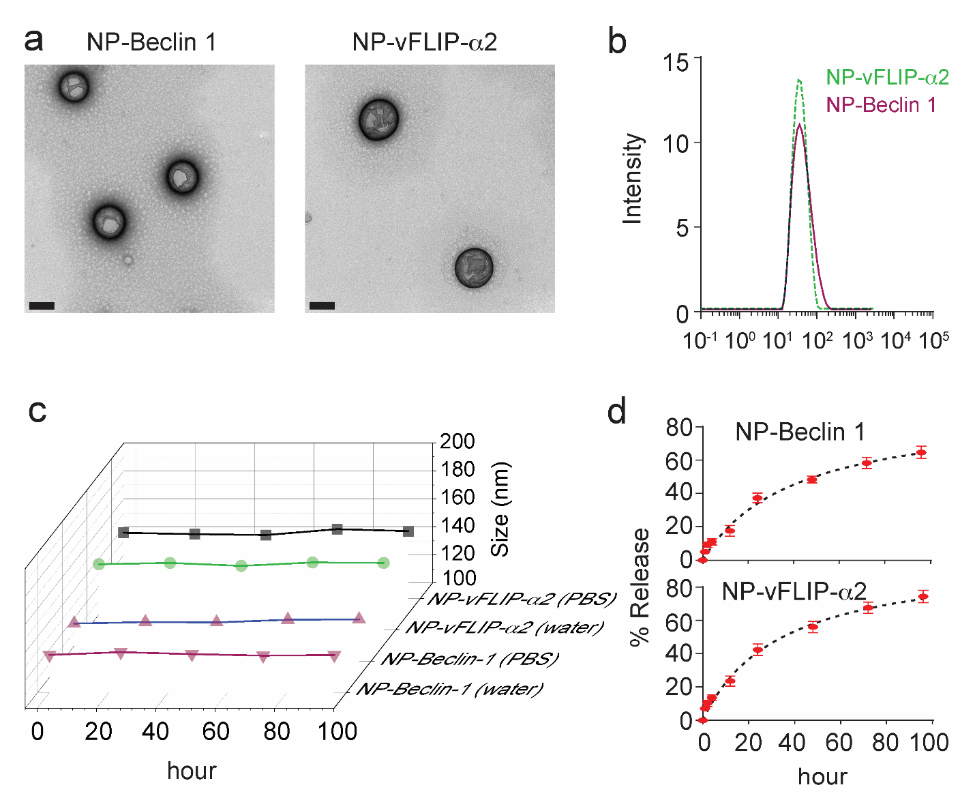
**

**Supplementary Fig. 2** Characterization of PLGA-lecithin-PEG nanoparticles loaded autophagy-inducing peptides. **a** The representative transmission electron microscopy images of NP-Beclin 1 and NP-vFLIP-α2 demonstrate the size and structure of the nanoformulated autophagy-inducing peptides. The scale bar equals 100 nm. **b** The hydrodynamic radius of the nanoparticles was calculated by the dynamic light scattering. **c** The stability of NP-Beclin 1 and NP-vFLIP-α2 was monitored over 96 h. **d** The controlled releasing kinetics of Tat-Beclin 1 and Tat-vFLIP-α2 was analyzed over 96 h.

**
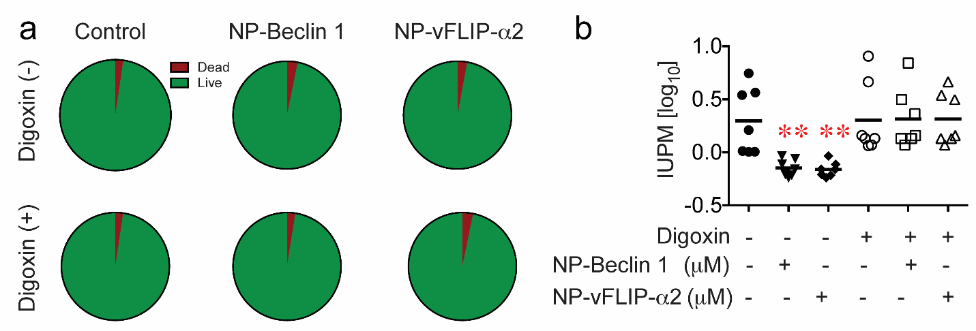
Supplementary Fig. 3** Na^+^/K^+^-ATPase inhibitor, digoxin, reverses NP-Beclin 1 and NP-vFLIP-α2 induced preferential kill of HIV latently infected cells *ex vivo*. **a** Purified resting CD4^+^ T cells from HIV-infected patients were pretreated with 50 nM digoxin for 2 h, and incubated with 10 μM NP-Beclin 1 or 10 μM NP-vFLIP-α2 for 24 h. Cell viability was quantified using trypan blue staining. **b** Replication competent virus from latently infected CD4^+^ T cells was quantified by QVOA assay. Data are summarized from 7 different donors and plotted with means. IUPM = infectious units per million resting CD4^+^ T cells, ** *P*<0.01.
